# SHP-1 activation inhibits vascular smooth muscle cell proliferation and intimal hyperplasia in a rodent model of insulin resistance and diabetes

**DOI:** 10.1007/s00125-016-4159-1

**Published:** 2016-12-09

**Authors:** Weier Qi, Qian Li, Chong Wee Liew, Christian Rask-Madsen, Samuel M. Lockhart, Lars Melholt Rasmussen, Yu Xia, Xuanchun Wang, Mogher Khamaisi, Kevin Croce, George L. King

**Affiliations:** 1 Research Division, Joslin Diabetes Center, Harvard Medical School, Dianne Nunnally Hoppes Laboratories, One Joslin Place, Boston, MA 02215 USA; 2 Department of Physiology and Biophysics, University of Illinois at Chicago, Chicago, IL USA; 3 Department of Clinical Biochemistry and Pharmacology, Center for Individualized Medicine in Arterial Diseases (CIMA), Odense University Hospital, Odense, Denmark; 4 Cardiovascular Clinical Research Center, Department of Medicine, Brigham and Women’s Hospital, Harvard Medical School, Boston, MA USA

**Keywords:** Diabetes, Insulin resistance, Restenosis, SHP-1

## Abstract

**Aims/hypothesis:**

Accelerated migration and proliferation of vascular smooth muscle cells (VSMCs) enhances arterial restenosis after angioplasty in insulin resistance and diabetes. Elevation of Src homology 2-containing protein tyrosine phosphatase 1 (SHP-1) induces apoptosis in the microvasculature. However, the role of SHP-1 in intimal hyperplasia and restenosis has not been clarified in insulin resistance and diabetes.

**Methods:**

We used a femoral artery wire injury mouse model, rodent models with insulin resistance and diabetes, and patients with type 2 diabetes. Further, we modulated SHP-1 expression using a transgenic mouse that overexpresses SHP-1 in VSMCs (*Shp-1*-Tg). SHP-1 agonists were also employed to study the molecular mechanisms underlying the regulation of SHP-1 by oxidised lipids.

**Results:**

Mice fed a high-fat diet (HFD) exhibited increased femoral artery intimal hyperplasia and decreased arterial SHP-1 expression compared with mice fed a regular diet. Arterial SHP-1 expression was also decreased in Zucker fatty rats, Zucker diabetic fatty rats and in patients with type 2 diabetes. In primary cultured VSMCs, oxidised LDL suppressed SHP-1 expression by activating *Mek-1* (also known as *Map2k1*) and increased DNA methylation of the *Shp-1* promoter. VSMCs from *Shp-1*-Tg mice exhibited impaired platelet-derived growth factor (PDGF)-stimulated tyrosine phosphorylation with a concomitant decrease in PDGF-stimulated VSMC proliferation and migration. Similarly, HFD-fed *Shp-1*-Tg mice and mice treated with the SHP-1 inducer, Icariside II, were protected from the development of intimal hyperplasia following wire injury.

**Conclusions/interpretation:**

Suppression of SHP-1 by oxidised lipids may contribute to the excessive VSMC proliferation, inflammatory cytokine production and intimal hyperplasia observed in arteries from diabetes and insulin resistance. Augmenting SHP-1 levels is a potential therapeutic strategy to maintain stent patency in patients with insulin resistance and diabetes.

**Electronic supplementary material:**

The online version of this article (doi:10.1007/s00125-016-4159-1) contains peer-reviewed but unedited supplementary material, which is available to authorised users.

## Introduction

A prominent feature in the pathology of restenosis and atherosclerosis is the increased number of vascular smooth muscle cells (VSMCs) in the intima of arteries due to enhanced VSMC migration and proliferation [1, 2]. The key role of abnormal VSMC growth in the pathogenesis of arterial restenosis is highlighted by the three- to fourfold reduction in restenosis rates with the use of drug-eluting stents that target smooth muscle cell proliferation and migration [3, 4]. However, even with the use of these stents, patients with diabetes still exhibit an increased risk of restenosis compared with non-diabetic patients [5].

Multiple mechanisms have been proposed to explain the excessive VSMC proliferation observed in diabetes and other insulin-resistant states. Hyperglycaemia and dyslipidaemia are thought to play important roles by increasing the expression of mitogenic growth factors [6]. Indeed, elevated expression of insulin, insulin-like growth factor-1 (IGF-1), platelet-derived growth factor (PDGF) and fibroblast growth factor-2 (FGF2) [7–9] has been associated with enhanced VSMC proliferation. However, there is disagreement in the literature about whether the expression of these growth factors is increased in the vascular wall or plasma in the context of diabetes [10]. Thus, it is still unclear if this mechanism drives excessive VSMC proliferation in the diabetic milieu.

In contrast, the possibility of enhanced action of growth factors through amplification of their intracellular signalling cascades has not been studied in detail. Src homology-2-containing protein tyrosine phosphatase (SHP-1) is a tyrosine phosphatase that functions as a negative regulator of growth-factor-dependent signalling. SHP-1 is a potent negative regulator of growth-factor signalling but the expression of SHP-1 and its contribution to vascular restenosis in animal models of obesity and type 2 diabetes have not been investigated.

In this report, we aimed to determine the expression level of SHP-1 in the arterial wall in animal models and human obesity and type 2 diabetes mellitus. Further, we studied the effect of oxidised lipids on SHP-1 expression in VSMCs and contribution to the excessive VSMC proliferation, inflammatory cytokine production and intimal hyperplasia observed in arteries leading to a high risk of restenosis in insulin resistance and diabetes. Finally, we aimed to determine whether pharmacologically augmenting SHP-1 expression could be an effective therapeutic approach to reduce restenosis.

## Methods

For detailed methods, please refer to the electronic supplementary materials (ESM) Methods.

### Animals and reagent

All protocols for animal use and euthanasia were reviewed and approved by the Animal Care Committee of the Joslin Diabetes Center. The experiments were in accordance with National Institutes of Health (NIH) guidelines following the standards established by the Animal Welfare Acts and by the documents entitled ‘Principles for Use of Animals’ and ‘Guide for the Care and Use of Laboratory Animals’. Male Zucker lean (ZL) rats, Zucker fatty (ZF) rats, Zucker diabetic fatty (ZDF) and their control (LEAN +/?) were from Charles River (Wilmington, MA, USA). C57BL/6 mice were from Jackson Laboratory (Bar Harbor, ME, USA). PDGF-BB was purchased from Sigma (St Louis, MO, USA).

### Procurement of human arteries

This work was approved by the regional ethics committee (Region Syddanmark, protocol number ID S-20100044). All patients over the age of 18 years, referred to coronary artery bypass grafting (CABG) surgery at Odense University Hospital, Denmark were asked to participate. Type 2 diabetes was defined as a history of diabetes, treatment with glucose-lowering medicine or HbA_1c_ > 6.5% and absence of IA2 and GAD65 autoantibodies. Arteries from a part of the internal thoracic artery were collected. Perivascular tissue was removed and arterial rings were fixed in formalin for paraffin embedding.

### Generation of SM22α-promoter-driven *Shp-1*-overexpressing transgenic mouse line

CMV promoter drives 1.57 kb Lox-stop-Lox (LSL) fragment followed by full-length *Shp-1* (also known as *Ptpn6*) cDNA cloned into Not1 and Xho1 (Fig. 3a). The final construct was digested with BstBI and Mlu and the 6.5 kb fragment was injected into blastocytes. LSL-*Shp-1* mice were crossbred with *Sm22*α-CreKI mice (stock no. 006878, Jackson Laboratory) to generate mice overexpressing *Shp-1* specifically in VSMCs, maintained on C57BL/6J background. Primers (see ESM Methods for details) were used for LSL-*Shp-1* genotyping.

### Femoral artery wire injury and histological analysis

Bilateral wire injury of the femoral artery was performed as previously described [11]. Mice were sacrificed on day 28 post arterial injury. Arteries were excised and paraffin-embedded. Sections (5 μm) were stained using Verhoeff tissue elastin stain (Sigma) and were masked for the measurement of the luminal, intimal and medial areas using NIH ImageJ (Bethesda, MD, USA).

### Mouse aortic smooth muscle cell culture

Aortas were dissected and digested with collagen II (Worthington, Columbia, NJ, USA). The adventitia was removed and further digested and grown in 20% DMEM. Cells at passage 2–3 and that had been starved for 48 h were used for all the experiments.

### VSMC transfection

Small interfering RNA for scramble or *Mek1* (also known as *Map2k1*) (sc-29396; Santa Cruz, Dallas, TX, USA) and *Jnk* (also known as *Mapk8*) (no. 6232; Cell Signaling Beverly, MA, USA) were transfected at 10 nmol/l using Basic Smooth Muscle Cells (SMC) Nucleofector Kit.

### Methylation analysis of the *Shp-1* promoter by bisulfite sequencing

Genomic DNA was extracted from VSMCs and bisulfite conversion was performed using EZ DNA Methylation Gold kit (Zymo Research, Irvine, CA, USA). The CT-converted DNA was then amplified (see ESM Methods for primer details). The PCR fragment was cloned into a TOPO vector for DNA sequencing. Methylation was analysed using BiQ Analyzer software (Max-Planck-Institut für Informatik, Saarbrücken, Germany).

### SHP-1 activity assay

SHP-1 was immunoprecipitated with 2 μg anti-SHP-1 polyclonal antibody (Santa Cruz) from control and *Shp-1*-Tg VSMCs overnight, 20 μl Protein A/G PLUS-Agarose (Santa Cruz) was added and beads were washed and then assayed by RediPlate 96 EnzChek Tyrosine Phosphatase Assay Kit (Molecular Probes, Eugene, OR, USA).

### Cell proliferation assays, cell cycle analysis and migration assays

Cell proliferation was measured using Click-iT EdU Flow Cytometry Assay Kit (Invitrogen, Carlsbad, CA, USA). The CellTiter 96 Non-Radioactive Cell Proliferation Assay (MTT assay; Promega, Madison, WI, USA) was used for the measurement of cell number. For cell cycle analysis, cells were and digested with RNase A. Propidium iodide was added before flow cytometry analysis. Migration was measured by Transwell insert using CytoSelect 24-Well Cell Migration Assay kit (Cell Biolabs, San Diego, CA, USA).

### Quantitative real-time PCR (qPCR) analysis

Total RNA was isolated and purified and converted into cDNA. *Shp-1*, *Shp-2* (also known as *Ptp11*), *Ptp1b* (*Ptpn1*), *Cyclin E1* (*Ccne1*), *Mcp-1* (*Ccl2*) and *36B4* (*Rplp0*). qPCR primers were used with SYBR Green Master mix kit and detection was performed using the ABI PRISM 7900 Sequence Detection System (Applied Biosystems, Foster City, CA, USA). Relative change was determined using the ΔΔ method and normalised to *36B4* (also known as *Rplp0*).

### Western blotting

Protein samples were electrophoresed in Bio-Rad TGX precast gels, transferred to a nitrocellulose membrane and incubated with primary antibodies (see ESM Methods for details). For p-Akt and Akt, and p-ERK and ERK (same molecular mass protein), two separate gels were used for phosphorylated and unphosphorylated protein instead of stripping the membrane to reprobe the other to avoid the inefficiency of stripping. Quantitative densitometry was performed using NIH Image J.

### Immunofluorescent staining

Mouse femoral arteries were fixed, embedded in paraffin and 5 μm sections were stained with antibodies (see ESM Methods for details). Photoshop (San Jose, CA, USA) was used for the quantification of SHP-1 expression levels in the media of arteries. For the Ki-67 and macrophage (MAC2) staining, positive cells with the overlay of Ki-67 or MAC2 (red) with smooth muscle cell marker (green) and DAPI (nucleus) were counted.

### Icariside II oral gavaging in HFD-fed mice

Icariside II (Syd Lab, Natick, MA, USA) was dissolved in 15% PEG 400 (Sigma) and given to mice fed a high-fat diet (HFD) via daily oral gavaging. 15% PEG 400 was given to vehicle-treated mice. Treatment started 1 day before femoral artery injury.

### Statistics

Comparison of two groups was made using unpaired *t* test. Comparison of more than two groups was performed by one-way ANOVA followed by the post hoc analysis with unpaired *t* test to evaluate statistical significance between the two groups. The data are presented as means ± SEM, unless otherwise stated. Statistical significance was defined as *p <* 0.05.

## Results

### HFD feeding enhances femoral artery intimal hyperplasia in response to wire injury

**Fig. 1:**
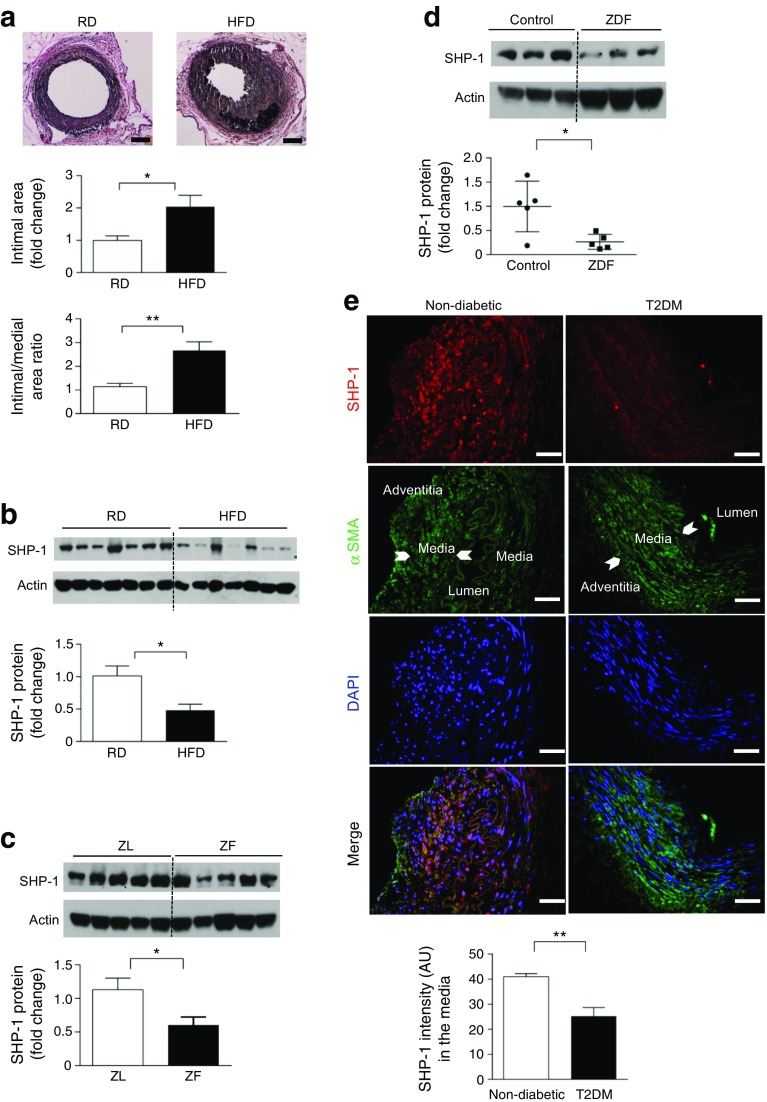
Insulin resistance and diabetes induce severe intimal hyperplasia in response to arterial injury and downregulate SHP-1 expression in rodent aortas and human arteries. (**a**) C57BL/6J mice were fed either RD or HFD for 4 weeks before injury was performed; *n* = 5–6; scale bar, 50 μm. (**b**) C57BL/6J mice were fed on either RD or HFD for 8 weeks; *n* = 7. (**c**, **d**) Aortas from ZF and ZDF rats at 14 weeks of age; *n* = 5. (**e**) Human mammary arteries; scale bar, 50 μm; *n* = 6. AU, arbitrary units; T2DM, type 2 diabetes. *p < 0.05 and **p < 0.01 for indicated comparison

To determine whether insulin resistance and diabetes increased intimal hyperplasia, wire injury in the femoral artery was performed on mice fed either a regular diet (RD) or an HFD (60% energy from fat) for 4 weeks starting from the age of 8 weeks. HFD-fed mice exhibited hypercholesterolaemia, obesity and insulin resistance as determined by measurement of plasma lipids and insulin levels and intraperitoneal insulin tolerance (IP-ITT) and glucose tolerance test (IP-GTT) (ESM Fig. 1a–c and ESM Table 1). Intimal hyperplasia in response to wire injury showed that the intimal area and intima/media ratio were increased by 100% and 133%, respectively, in HFD-fed mice compared with RD-fed mice (Fig. 1a).

### Insulin resistance and diabetes downregulates SHP-1 expression in rodent aorta and human arteries

Expression of SHP-1 in the aorta was measured in three models of obesity, insulin resistance and type 2 diabetes, including mice with diet-induced obesity, ZF rats and ZDF rats. SHP-1 protein was significantly decreased in aortas from HFD-fed mice, ZF rats and ZDF rats compared with their respective controls (Fig. 1b–d). In contrast, expression of other protein tyrosine phosphatases (PTPs) PTP1B and SHP-2 was not changed in the femoral arteries from HFD vs. RD mice or in the neointima and media of injured femoral artery (ESM Fig. 1d, e). Thus, SHP-1, but not other PTPs, is decreased in insulin resistance and diabetes. SHP-1 protein levels were also significantly decreased in the media of mammary arteries from patients with type 2 diabetes compared with non-diabetic patients (Fig. 1e) as assessed by double immunostaining of SHP-1 and α-smooth muscle actin (α-SMA). Clinical characteristics of the patients with type 2 diabetes included elevated levels of HbA_1c_, total cholesterol, LDL and triacylglycerol levels when compared with individuals without type 2 diabetes (ESM Table 2).

### Effect of lipids and glucose on SHP-1 expression and its mechanisms

**Fig. 2:**
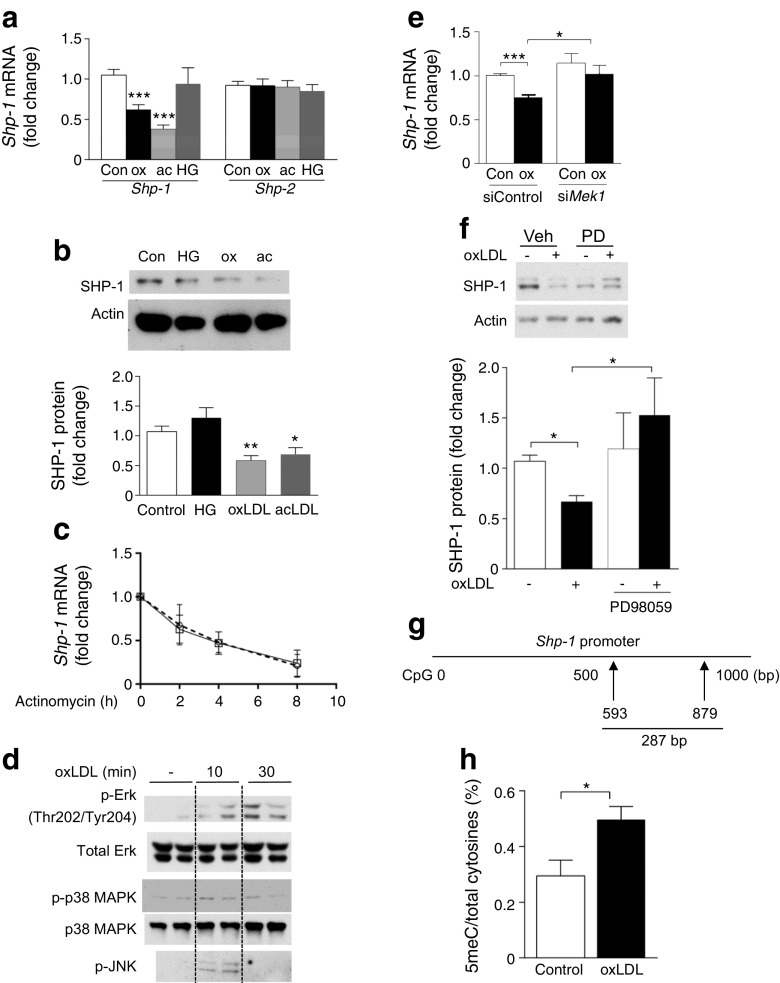
oxLDL decreases SHP-1 expression in VSMCs via elevating DNA methylation on the *Shp-1* promoter. (**a**, **b**) VSMCs were starved for 24 h in 0.1% fatty-acid-free BSA control, or treated with 100 μg/ml oxLDL (ox), 100 μg/ml acLDL (ac) or 25 mmol/l d-glucose (HG) for 8 h. *Shp-1* mRNA (**a**) and protein (**b**) were analysed; *n* = 3 and 5, respectively. (**c**) VSMCs were treated with 5 μg/ml actinomycin with or without oxLDL; *n* = 3; squares, 0.1% BSA; circles, oxLDL. (**d**) VSMCs treated with control and oxLDL; *n* = 3. (**e**) VSMCs transfected with 10 nmol/l scramble (siControl) or *Mek1* small interfering RNA (si*Mek1*) and treated with oxLDL. (**f**) VSMCs treated with control and oxLDL in the presence or absence of 10 μmol/l PD98059 (PD) for 24 h; *n* = 5. (**g**, **h**) Bisulfite sequencing at the region of *Shp-1* promoter. Data are presented as the ratio of unconverted cytosine (5-methylcytosine, 5meC) and total cytosines. *p < 0.05, **p < 0.01 and ***p < 0.001 vs control or for indicated comparison

As HFD mice and ZF and ZDF rats exhibited hyperlipidaemia, hyperglycaemia and hyperinsulinemia (ESM Table 1 and ESM Fig. 1a–c), we evaluated the effects of modified lipids and hyperglycaemia on the regulation of SHP-1 expression in mouse aortic VSMCs in vitro. In primary cultures of VSMCs from C57BL/6J mice, the expression of *Shp-1* mRNA (Fig. 2a) and protein (Fig. 2b) was decreased by oxidised LDL (oxLDL) and acetyl-LDL (acLDL). No change in SHP-1 expression was observed when VSMCs were incubated in high (25 mmol/l) vs. low (5.5 mmol/l) glucose conditions (Fig. 2a, b) or were treated with insulin (ESM Fig. 2a, b). In the presence of actinomycin D, the half-life of *Shp-1* mRNA in VSMCs was 4 h and was not changed by treatment with oxLDL (Fig. 2c). Since oxLDL activates several stress kinases, we studied the effect of oxLDL on the activation of mitogen-activated protein kinase (MAPK)/extracellular signal-regulated kinase (ERK) (MEK), p38MAPK and c-Jun N-terminal kinase (JNK). All three were activated by oxLDL (Fig. 2d). However, only silencing *Mek1* impaired the ability of oxLDL to suppress SHP-1 expression (Fig. 2e, f and ESM Fig. 3a); we did not observe any change in VSMCs with p38αMAPK, p38βMAPK or JNK knockdown (ESM Fig. 3b–e). Prior study has suggested downregulation of SHP-1 expression could be mediated by increased DNA methylation of its promoter in cancer cells [12]. To delineate the mechanism underlying regulation of SHP-1 by oxLDL, we studied the effect of oxLDL treatment on methylation at the *Shp-1* promoter. Bisulfite sequencing was performed on the region of the *Shp-1* promoter ranging between +593 and +879 relative to the +1 transcription start site of the *Shp-1* gene. We observed a 68% increase in unconverted cytosines on the *Shp-1* promoter in VSMCs treated with oxLDL vs control (Fig. 2g, h), suggesting that DNA methylation was increased in the *Shp-1* promoter. Thus, the expression of SHP-1 is decreased in diabetes potentially via oxLDL induced-methylation of the *Shp-1* promoter.

### Generation and characterisation of *Shp-1*-Tg mice

**Fig. 3:**
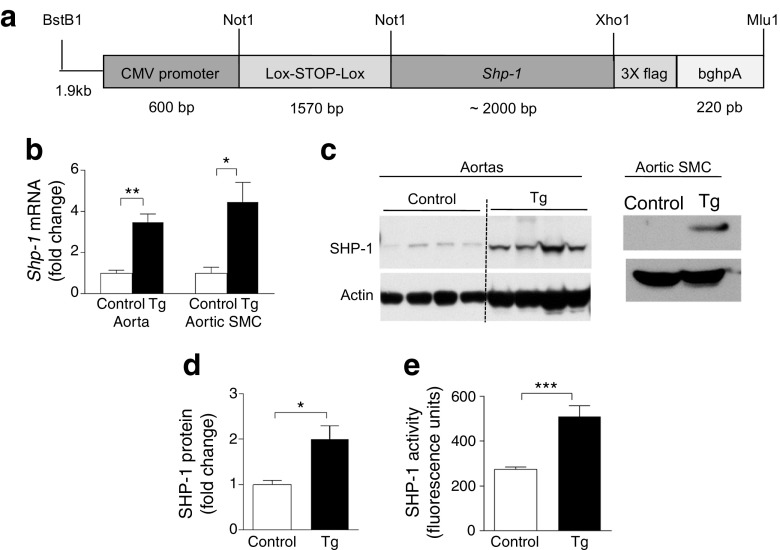
Generation and characterisation of *Shp-1*-Tg mice. (**a**) Schema of LSL-*Shp-1* mice crossbred with *Sm22α*-CreKI mice to generate mice overexpressing *ShP-1* specifically in VSMCs. (**b**–**e**) SHP-1 mRNA and protein from aortas and aortic SMCs from control and *Shp-1*-Tg mice; *n* = 3–5. Tg, *Shp-1*-Tg mice. *p < 0.05, **p < 0.01 and ***p < 0.001 for indicated comparison

To support the idea that enhancing SHP-1 expression in VSMCs can decrease intimal hyperplasia in insulin resistance and type 2 diabetes, we generated transgenic mice overexpressing SHP-1 specifically in VSMCs (Fig. 3a). *Shp-1* mRNA and protein levels were increased more than twofold in aortas and VSMCs of *Shp-1*-Tg mice compared with control mice (Fig. 3b–d). Further, SHP-1 activity was increased twofold in VSMCs from *Shp-1*-Tg mice compared with control mice (Fig. 3e). In contrast, mRNA expression of other PTPs, *Ptp1b* and *Shp-2*, was not changed in VSMCs from *Shp-1*-Tg mice vs. control mice (ESM Fig. 4a).

### SHP-1 overexpression inhibits the activation of PDGF, insulin and FGF2 receptors and their downstream signalling in VSMCs

**Fig. 4:**
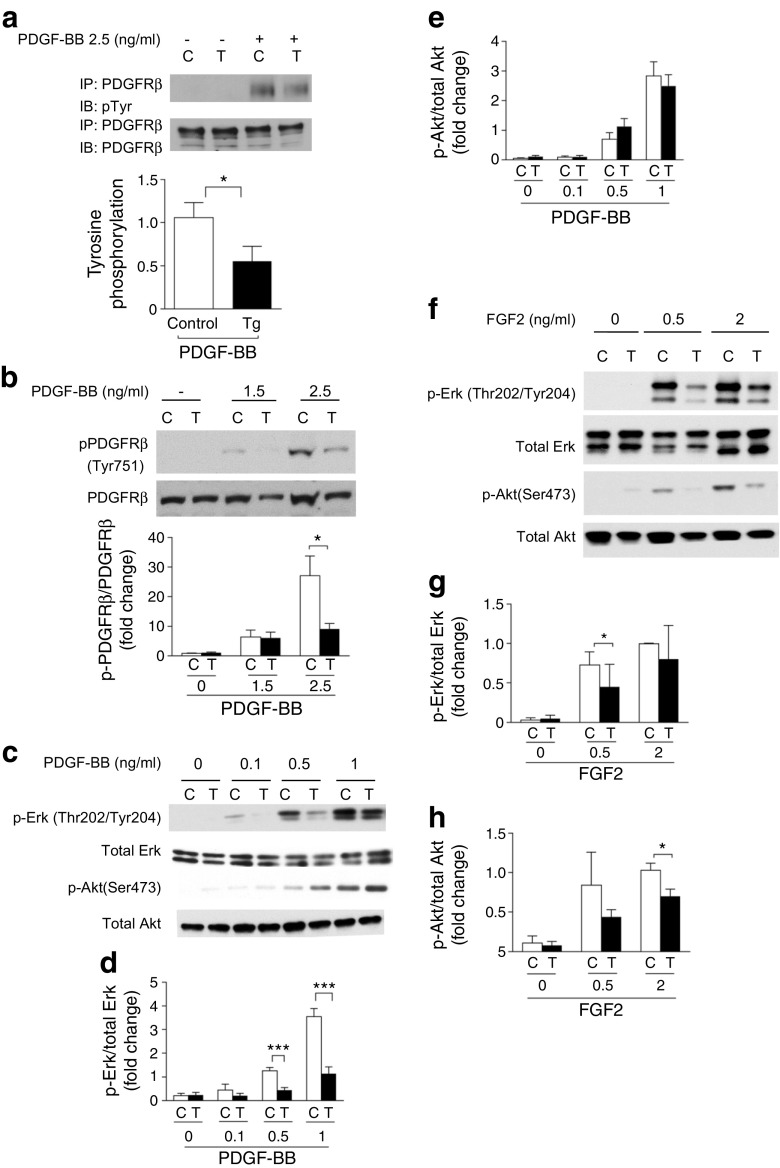
SHP-1 overexpression inhibits the activation of PDGFRβ and FGF2 receptors and their downstream signalling in VSMCs. (**a**) VSMCs stimulated with 2.5 ng/ml PDGF-BB for 5 min. *n* = 4 per group. (**b**) Tyr751 stimulated with PDGF-BB for 5 min; *n* = 4. (**c**–**e**) VSMCs stimulated with PDGF-BB for 5 min; *n* = 4. (**f**–**h**) VSMCs stimulated with FGF2 for 5 min. C, VSMCs from control mice; T, VSMCs from *Shp-1*-Tg mice; IB, immunoblot; IP, immunoprecipitation; *n* = 3. *p < 0.05 and ***p < 0.001 for indicated comparisons

The mitogenic actions of growth factors such as PDGF, FGF2 and insulin can affect VSMC migration and proliferation [13–15]. These factors signal via receptor tyrosine kinases and their actions may be suppressed by SHP-1. Thus, we determined whether overexpression of SHP-1 inhibited the activation of tyrosine kinase receptors. First, in VSMCs isolated from *Shp-1*-Tg mice tyrosine phosphorylation of PDGF receptor- β (PDGFRβ) was significantly decreased in response to PDGF-BB (Fig. 4a). We then determined whether SHP-1 targets tyrosine phosphorylation at a specific site of on PDGFRβ. Three tyrosine phosphorylation sites (Tyr751, Tyr740, Tyr771) were reported to be stimulated by PDGF-BB [16]. Tyr751 was significantly decreased in VSMCs from *Shp-1*-Tg mice vs. control mice in response to PDGF-BB (Fig. 4b). No difference was observed at Tyr740 or Tyr771 (ESM Fig. 4b,c). Thus, SHP-1 inhibits the action of PDGF-BB via targeting Tyr751. Activation of p-ERK by PDGF-BB (0.1 and 0.5 ng/ml) was decreased significantly in VSMCs from *Shp-1*-Tg mice (Fig. 4c, d). Interestingly, no difference was observed in Akt activation (p-Akt) between control mouse and *Shp-1*-Tg mouse VSMCs at basal or with PDGF stimulation (Fig. 4e).

FGF2 signalling in VSMCs was also studied because activated platelets secrete FGF2, resulting in VSMC migration and proliferation during restenosis [14]. Both p-ERK and p-Akt levels induced by FGF2 were significantly decreased in VSMCs from *Shp-1*-Tg mice (Fig. 4f–h). Moreover, overexpression of SHP-1 also decreased insulin receptor β tyrosine phosphorylation and p-ERK activation in response to insulin but insulin-stimulated increases in p-Akt were not affected (ESM Fig. 4d–f).

### Overexpression of SHP-1 in VSMCs inhibits the activation of ERK and Akt in vivo

The effect of overexpressing SHP-1 on p-ERK and p-Akt activation in vivo was studied in control mice and *Shp-1*-Tg mice fed with HFD for 8 weeks before femoral artery wire injury. No significant differences in body weight, blood pressure, glucose tolerance, insulin tolerance or plasma lipid concentrations between control and *Shp-1*-Tg mice on HFD were observed (ESM Fig. 5a–g). Further, we did not observe any difference in *Shp-1* mRNA expression between the sham and injury groups (ESM Fig. 6).

**Fig. 5:**
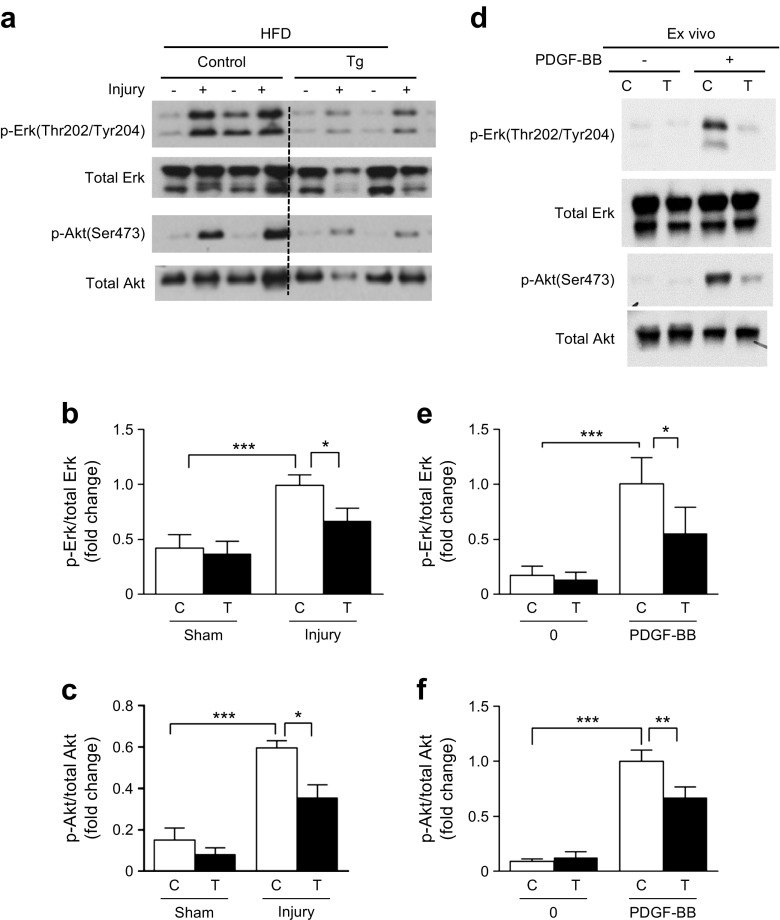
Overexpression of SHP-1 in VSMCs inhibits the activation of ERK and Akt in vivo in response to arterial injury in mice fed an HFD. (**a**–**c**) Mice on HFD for 8 weeks at 1 h post injury. (**d**–**f**) Aortas incubated in 100 ng/ml PDGF-BB for 10 min. C, control mice, T, *Shp-1*-Tg mice. *n* = 5. *p < 0.05, **p < 0.01 and ***p < 0.001 for indicated comparisons

After 8 weeks on HFD, basal levels of p-ERK were similar in control and *Shp-1*-Tg mice with sham injury (Fig. 5a, b). In contrast, wire injury increased p-ERK expression in the femoral artery twofold compared with control mice with sham injury; this effect was significantly decreased in injured femoral arteries from *Shp-1*-Tg mice (Fig. 5b). In addition, p-Akt activation in *Shp-1*-Tg and control mice on HFD after wire injury showed a fourfold increase; this was significantly inhibited in *Shp-1*-Tg mice (Fig. 5c). Similarly, PDGF signalling was enhanced in whole aortas from *Shp-1*-Tg mice stimulated with PDGF-BB ex vivo (Fig. 5d–f).

### Inhibition of cell proliferation, cell cycle progression and migration in VSMCs from *Shp-1*-Tg mice

**Fig. 6:**
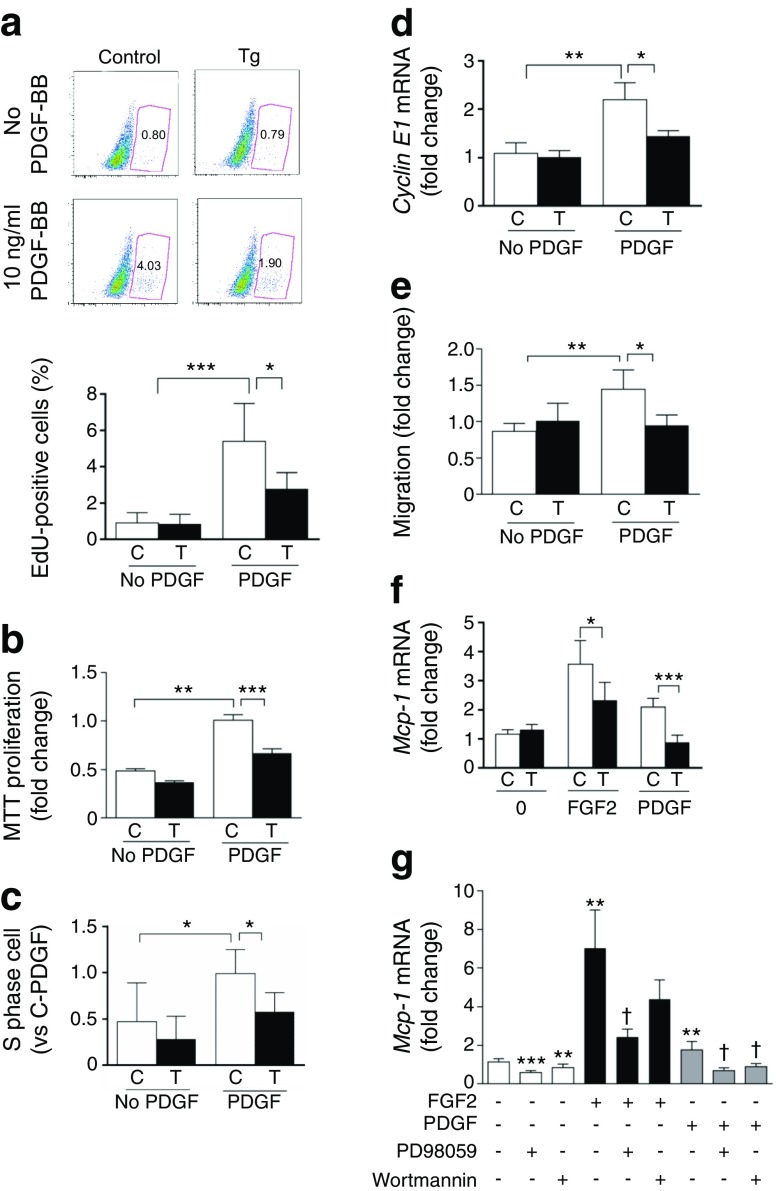
Overexpression of SHP-1 in VSMCs inhibits proliferation, cell cycle progression and migration. (**a**) VSMCs stimulated with or without 10 ng/ml PDGF-BB for 16 h; *n* = 4. (**b**) VSMCs with or without PDGF-BB (PDGF) for 48 h; *n* = 4. (**c**) VSMCs stimulated with or without PDGF-BB for 24 h; *n* = 5. C-PDGF, control VSMCs treated with PDGF. (**d**) *Cyclin E1* mRNA; *n* = 4. (**e**) VSMCs stimulated with or without PDGF-BB for 8 h. Migrated cells measured by Transwell; *n* = 4. (**f**) VSMCs stimulated with or without 10 ng/ml FGF2 or PDGF-BB for 8 h; *n* = 3. (**g**) VSMCs pre-treated with 50 μmol/l PD98059 or 100 nmol/l wortmannin for 30 min and co-incubated with FGF2 or PDGF-BB for 8 h; *n* = 4. C, control VSMCs; T, *Shp-1*-Tg VSMCs. EdU, ethynyldeoxyuridine; MTT, 3-(4,5-dimethyl-2-thiazolyl)-2,5-diphenyl-2*H*-tetrazolium bromide; *p < 0.05, **p < 0.01 and ***p < 0.001 for indicated comparisons (or vs no treatment in **g**); ^†^ *p <* 0.05 vs PDGF-BB vehicle or FGF2 vehicle

PDGF-BB increased cell proliferation in VSMCs from control mice as measured by BrdU incorporation and MTT assay. This effect was reduced in VSMCs from *Shp-1*-Tg mice (Fig. 6a, b and ESM Fig. 7a). Cell cycle analysis showed that PDGF-BB-induced cell cycle progression from G1 to S phase was inhibited by SHP-1 overexpression in VSMCs (Fig. 6c). In keeping with these findings, PDGF-BB-induced cyclin E1 expression was decreased in VSMCs from *Shp-1*-Tg mice (Fig. 6d). In contrast, the rate of VSMC apoptosis in *Shp-1*-Tg mice was no different from that in control mice (ESM Fig. 7b). We also assessed whether overexpression of SHP-1 could affect VSMC migration in response to PDGF-BB. Migration assays showed that PDGF-induced migration was reduced significantly in VSMCs from *Shp-1*-Tg mice compared with control mice (Fig. 6e). Together, our findings demonstrate that SHP-1 overexpression impairs mitogenic signalling and limits growth-factor-induced proliferation and migration of VSMCs.

### Overexpression of SHP-1 in VSMCs inhibits the expression of inflammatory cytokine monocyte chemoattractant protein-1

The number of inflammatory cells in the arterial wall after injury may contribute to the severity of the restenosis process [17]. Therefore, we characterised the effect of PDGF-BB and FGF2 on the expression of monocyte chemoattractant protein-1 (MCP-1) in VSMCs. FGF2 and PDGF-BB increased *Mcp-1* mRNA levels in VSMCs from control mice but this induction was relatively impaired in VSMCs from *Shp-1*-Tg mice (Fig. 6f). Mechanistically, a selective inhibitor of the p-ERK pathway, PD98059, significantly inhibited the effect of FGF2 and PDGF-BB on *Mcp-1* mRNA expression. The addition of wortmannin, an inhibitor of the phosphoinositide 3-kinase/Akt pathway, impaired the ability of PDGF-BB to induce MCP-1 expression but had no effect on FGF2-dependent increases in MCP-1 (Fig. 6g).

### Reduction of intimal hyperplasia in response to injury in HFD-fed *Shp-1*-Tg mice 

**Fig. 7:**
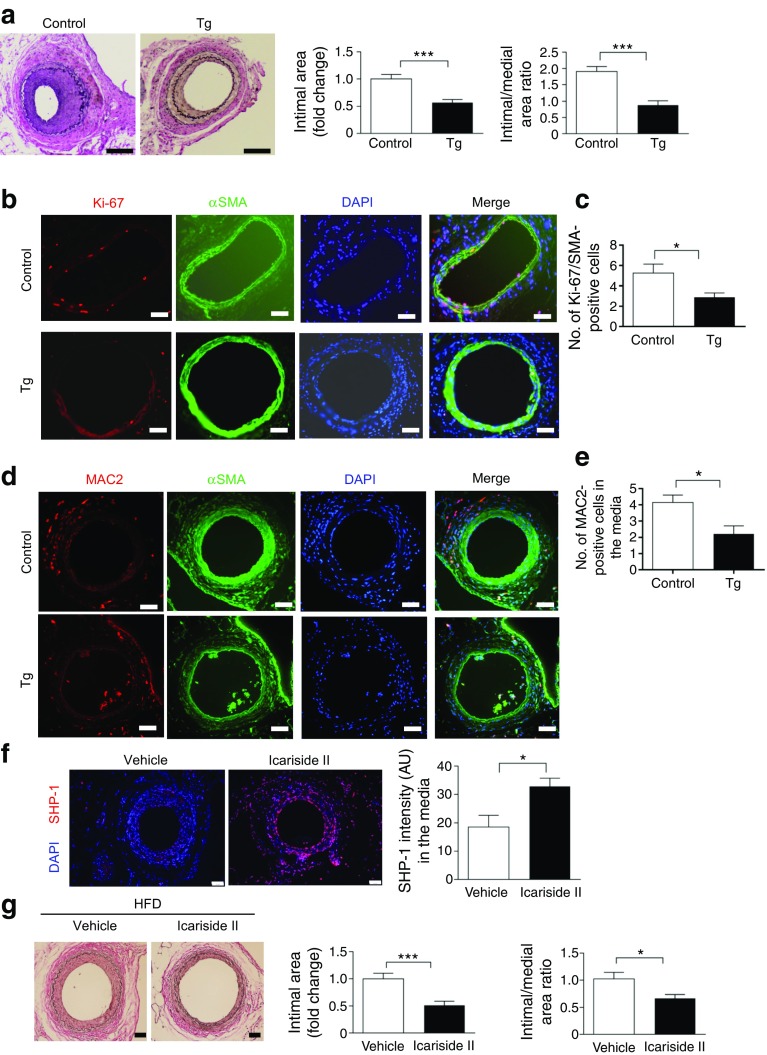
HFD-fed mice overexpressing SHP-1 develop less intimal hyperplasia and exhibit a decrease in cell proliferation and macrophage content in the vascular wall lesions in response to injury. (**a**) Mice were fed HFD for 8 weeks and examined at day 28 post injury; *n* = 7–10, scale bar, 100 μm. (**b–e**) Mice were fed HFD for 8 weeks and examined at day 7 post injury. The number of positive Ki-67 and αSMA were counted; *n* = 8, scale bar, 20 μm (**b**, **c**). The number of positive MAC2 in the media was counted; *n* = 5 arteries per group; scale bar, 20 μm (**d**, **e**). (**f**, **g**) Mice were fed HFD for 8 weeks and 100 mg/kg Icariside II was administered 1 day before injury via daily oral gavaging for 28 days. *n* = 4–6 arteries, scale bar, 50 μm (**f**). (**g**) Intimal area and intimal–medial ratio were quantified and analysed; *n* = 13–16, scale bar, 20 μm. *p < 0.05 and ***p < 0.001 for indicated comparisons

To determine further the causal relationship between decreased SHP-1 expression and accelerated intimal hyperplasia in insulin resistance and diabetes, we studied the severity of intimal hyperplasia in response to wire injury in the femoral artery from *Shp-1*-Tg and control mice after 8 weeks of HFD feeding. The intimal area measured at 28 days after injury was reduced by 43% in *Shp-1*-Tg mice compared with control mice. The ratio of intimal to medial area also exhibited a reduction of 54% in *Shp-1*-Tg compared with control mice on HFD (Fig. 7a).

### Cell proliferation and macrophage content in the vascular wall in response to injury

To confirm that the suppression of proliferation and pro-inflammatory cytokine expression observed in SHP-1-overexpressing VSMCs mediated the protective action of SHP-1 in arterial injury, we studied VSMC proliferation and macrophage number in the wall of the femoral artery 8 days after wire injury. Proliferating smooth muscle cells in the injured artery, as determined by staining α-SMA and Ki67, were counted in the media and neointima. Double-immunostained cells were significantly less abundant in *Shp-1*-Tg mice than in control mice (Fig. 7b, c). Furthermore, the macrophage content of media, as quantified by immunostaining for MAC2, was significantly decreased in the media of the femoral arteries from *Shp-1*-Tg mice compared with control mice after injury (Fig. 7d, e).

### Reduction of intimal hyperplasia in response to injury in HFD mice treated with SHP-1 activator

Icariside II was reported to increase SHP-1 expression and have an antiproliferative effect in cancer cells [18]. Further, Icariside II exhibited a reasonable pharmacokinetics profile in a single dose oral gavaging study [19]. Thus, Icariside II was given 1 day before mice underwent arterial injury at a dose of 100 mg/kg via daily oral gavaging for 28 days. In line with previous studies [18], we observed that SHP-1 expression was increased in the femoral arteries of Icariside II-treated mice when compared with mice treated with vehicle (Fig. 7f). Notably, we observed significant reduction in intimal area and the ratio of intimal to medial area at 28 days after injury in HFD-fed mice treated with Icariside II when compared with HFD-fed mice treated with vehicle (Fig. 7g).

## Discussion

The results from this study demonstrate that SHP-1 expression in arterial VSMCs is decreased in insulin-resistant and diabetic rodents and in patients with type 2 diabetes. In addition, overexpression of SHP-1 in VSMCs, induced by both genetic and pharmacological approaches, protected against intimal hyperplasia in response to arterial wire injury by inhibiting growth-factor-dependent signalling.

The mitogenic effects of diabetes and insulin resistance on VSMCs in both restenosis and atherosclerosis have always been puzzling considering the increases in apoptosis observed in many other vascular cells including retinal pericytes, podocytes and even endothelial cells [20–22]. Interestingly, we previously demonstrated that diabetes and hyperglycaemia upregulate the expression of SHP-1 in vascular contractile cells from the capillaries of the retina and glomeruli [20, 22]. However, in the current study hyperglycaemia had no effect on SHP-1 expression in arterial VSMCs, whereas modified lipids suppressed SHP-1 expression. Thus, these findings identify differential regulation of SHP-1 by hyperglycaemia and lipids in the micro- and macrovasculature as a potential mechanistic explanation for the dichotomous pathologies of contractile cells in macro- and microvessels in diabetes [20, 23].

Dyslipidaemia rather than dysglycaemia seems to drive the changes in SHP-1 expression in animal models of obesity and diabetes as in vitro studies demonstrated that treatment with modified lipids, but not hyperglycaemia suppressed SHP-1 expression. The pathogenic effects of hyperlipidaemia on cells are mainly mediated by modified LDL such as oxLDL and acLDL. We found that both oxLDL and acLDL inhibited SHP-1 expression indicating that decreased SHP-1 expression in hyperlipidaemic status is not oxLDL-specific and another modified LDL could also be involved. Further studies will be needed to confirm that these findings are relevant to human diseases. However, our finding, in a limited cohort, that SHP-1 expression is suppressed in type 2 diabetes patients is encouraging.

Overexpression of SHP-1 in VSMCs from RD-fed mice decreased the mitogenic action of several growth factors including PDGF-BB, FGF2 and insulin. SHP-1 overexpression exerts this action via inhibition of tyrosine phosphorylation at the receptor level via Tyr751 on PDGFRβ, thus inhibiting activation of p-ERK and preventing growth-factor-induced cell cycle progression. It has been reported that the phosphorylation of Tyr751 is required for both phosphoinositide 3-kinase and GTPase-activating protein (GAP) binding in epithelial cells [16, 24]. Further studies are needed to determine the mechanistic significance of the reduction in Tyr751 of PDGFRβ in VSMCs.

Our study is consistent with previous findings of the inhibitory effect of SHP-1 on the ERK pathway [25] and PDGFR signalling [26] in VSMCs. The activation of p-Akt in response to PDGF-BB and insulin stimulation of VSMCs was not affected by SHP-1 overexpression. In contrast, stimulation by PDGF in ex vivo experiments showed that the signalling of both p-Akt and p-ERK were diminished and that SHP-1 overexpression could significantly reduce p-Akt and p-ERK induction by arterial injury. It is possible that the in vivo effect of SHP-1 alters the action of multiple growth factors in the Akt pathway. Interestingly, potentiation of phosphatases upstream of Akt have previously been suggested as a therapeutic strategy in the context of vascular injury and restenosis [27]. Our work validates this suggestion in the context of diabetes and obesity. Elucidation of the role of regulatory phosphatases other than SHP-1 may allow us to fine tune the function of vascular mitogens and optimise growth-factor-dependent signalling in response to vascular injury [27–29].

Importantly, induction of SHP-1 overexpression in VSMCs using both genetic and pharmacological approaches was sufficient to normalise the response to wire injury in mice with diet-induced obesity. These results indicate that therapeutic intervention to induce gain of SHP-1 function or normalisation of SHP-1 levels in stents could decrease restenosis in diabetes and obesity. While SHP-1 is a potential therapeutic target, translational challenges exist. Kappert’s group have shown that systemic administration of an SHP-1 inhibitor improves insulin sensitivity in animal models of diet-induced obesity [30]. Moreover, HFD increases SHP-1 in a number of tissues [23]. Thus, future therapeutic strategies to activate SHP-1 and prevent vascular restenosis should consider targeted delivery of SHP-1 modulators either via topical application in the form of drug-eluting stents or cell-specific delivery using targeted delivery strategies such as ligand-conjugated nanoparticles [31].

In summary, our findings suggest a new perspective on how diabetes and insulin resistance increase the proliferation and migration of VSMCs. oxLDL-mediated downregulation of SHP-1 enhances growth-factor signalling in insulin resistance and diabetes and enhances intimal hyperplasia in response to arterial injury. These results support manipulation of SHP-1 activity or expression as a novel therapeutic approach to prevent restenosis and improve angioplasty outcomes in patients with insulin resistance and diabetes.

## Electronic supplementary material

Below is the link to the electronic supplementary material.

ESM (PDF 1107 kb)

## Abbreviations

acLDL: Acetyl-LDL
ERK: Extracellular signal-regulated kinase
FGF2: Fibroblast growth factor-2
HFD: High-fat diet
JNK: c-Jun N-terminal kinase
MAPK: Mitogen-activated protein kinase
MCP-1: Monocyte chemoattractant protein-1
MEK: MAPK/ERK
oxLDL: Oxidised LDL
PDGF: Platelet-derived growth factor
PDGFR: PDGF receptor
PTP: Protein tyrosine phosphatase
qPCR: Quantitative real-time PCR
RD: Regular diet
α-SMA: α-Smooth muscle actin
VSMC: Vascular smooth muscle cell
