# Supplementary material for: SHP-1 activation inhibits vascular smooth muscle cell proliferation and intimal hyperplasia in a rodent model of insulin resistance and diabetes
Source: Diabetologia. 2016 Dec 9;60(3):585–96. doi: 10.1007/s00125-016-4159-1 (PMC5672905; doi:10.1007/s00125-016-4159-1)
Supplement: Supplementary file 1 — (PDF 1107 kb) [file 125_2016_4159_MOESM1_ESM.pdf]

ESM method

Methods

Animal studies and reagent

All protocols for animal use and euthanasia were reviewed and approved by the Animal Care Committee of the Joslin Diabetes Center. They are in accordance with NIH guidelines following the standards established by the Animal Welfare Acts and by the documents entitled “Principles for Use of Animals” and “Guide for the Care and Use of Laboratory Animals”.

Male Zucker lean (ZL), Zucker Fatty rats (ZF), Zucker diabetic fatty (ZDF) and their control (LEAN +/?) were from Charles River (Wilmington, MA, USA). C57BL/6 mice were from JAX (Bar Harbor, ME, USA). High-fat diet 60% kcal of fat was from Research Diets (New Brunswick, NJ, USA), oxidized- and acLDL from Biomedical Technologies (Stoughton, MA, USA), and Icariside II was from Syd Labs (Natick, MA, USA). SHP-1, actin, PDGFRβ, insulin receptor were from Santa Cruz, and SHP-2, phospho-Akt, phospho Erk, Akt, Erk, PDGFRβ, insulin receptor β and phospho SAPK/JNK were from Cell Signalling.

Femoral artery wire injury and histological analysis

Bilateral wire injury of the femoral artery was performed as previously described [11]. Mice were harvested post-28 day arterial injury. Arteries were excised and paraffin-embedded. 5 μm sections were stained using Verhoeff tissue elastin stain (Sigma, St. Louis, MO, USA). Sections were masked for the measurement of the luminal, intimal, and medial areas using NIH ImageJ.

Procurement of human arteries

Protocol covering this work was submitted to the Danish Data Protection Agency (Datatilsynet) and was approved by the regional ethical committee (Region Syddanmark, protocol number ID S-20100044). All patients referred to CABG surgery at Odense University Hospital, Denmark were asked to participate. Inclusion criteria were age 18 years or older, planned CABG and informed consent. Type 2 diabetes was defined as a history of diabetes or treatment with anti-diabetic medicine or HbA1c>6.5% in combination with absence of IA2 and GAD65 autoantibodies. During coronary artery bypass (CABG) surgery, the remaining arteries from a part of the internal thoracic artery (ITA) used for the bypass graft were collected. Perivascular tissue was removed and arterial rings were fixed in formalin for paraffin embedding.

Mouse aortic smooth muscle cell culture

Aortas were dissected and digested with collagen II (Worthington, Columbia, NJ, USA). Adventitia was removed and further digested in collagen I. The digested mixture was grown in 20% DMEM medium. Cells at passage 2–3 were used for all the experiments. Cells were starved in DMEM medium containing 0.1% BSA for 48 h before experiments.

VSMC transfection

siRNAs for scramble or *Mek1* (Santa Cruz, sc-29396) and *Jnk* (Cell Signaling, #6232) were transfected at 10nM concentration using Basic Smooth Muscle Cells (SMC) Nucleofector® Kit. Transfection efficiency was monitored by GFP and the

expression levels of genes interested.

Methylation analysis of the *Shp-1* promoter by bisulfite sequencing
Genomic DNA was extracted from VSMC and bisulfite conversion was performed using EZ DNA Methylation Gold kit (Zymo Research). The CT converted DNA was then amplified using KAPA HiFi HotStart Uracil+ ReadyMix PCR Kit (Kapa Biosystems) with the following primers: sense 5'-GGTTAGGGTTATTTTTTGGGTTTTGTTTT-3'; antisense 5'-CTTAACCACTTCCTCCTCTATCCAACCTAT-3'. PCR fragment was subsequently cloned into TOPO vector (TOPO TA Cloning Kit) for sequencing. Cell culture was replicated twice and 8-11 clones from each group were sequenced. Data are presented as the ratio of unconverted cytosine (5-Methylcytosine, 5meC) and total cytosines Methylation site is analyzed using BiQ Analyzer software.

Generation of SM22α-promoter driven *Shp-1* overexpressing transgenic mouse line
CMV promoter drives 1.57kb Lox-stop-Lox fragment followed by a full length *Shp-1* cDNA, which was cloned into Not1 and Xho1 (Fig. 3a). The final construct was digested with BstBI & Mlu and the 6.5-kb fragment was injected into blastocytes. LSL-SHP1 mice were crossbred with SM22α-CreKI mice (stock #006878, Jackson Lab) to generate VSMC specific SHP-1 overexpression mice. This transgenic line is maintained on C57BL/6J background. The following primers were used for LSL-*Shp-1* genotyping: 5'-TAGCAAGAGCAAGAAGGAAGAG-3' and 5'-GGGGCAAACAACAGATGGCTG-3'; SM22α-CreKI cre genotyping: 5'- GGC CCA GGG GTT GTC AAA ATA GTC -3', 5'- CTC CTC CAG CTC CTC GTC ATA CTT C-3' and 5'- CGC CGC ATA ACC AGT GAA ACA G-3'.

SHP-1 activity assay
SHP-1 was immunoprecipitated with 2 μg anti-SHP-1 polyclonal antibody (Santa Cruz Biotechnology, Inc.) from Control and SHP-1Tg VSMC overnight. 20 ul Protein A/G PLUS-Agarose (Santa Cruz Biotechnology, Inc.) was added and incubated for 2 hrs at 4°C. Beads were washed three times with PBS containing 1% NP-40 and 5 mM DTT. Beads were then assayed by RediPlate 96 EnzChek Tyrosine Phosphatase Assay Kit (Molecular Probes, Eugene, OR, USA) as per manufacturer's instructions.

Cell proliferation assays, cell cycle analysis and migration assays

Cell proliferation and apoptosis assays Cell proliferation was measured using Click-iT® EdU Flow Cytometry Assay Kit (Invitrogen). Cells were seeded at a density of 100,000 cells per well on a 6-well plate and starved on day two in DMEM medium containing 0.1% BSA for 48 hours. Cells were then stimulated with 10ng/ml PDGF-BB for 16 hours and EdU was added for 8 hours. Cells were then fixed and labeled as per manufacturer's instructions. EdU positive cells were quantified by flow cytometer (LSR II, BD Biosciences). BrdU Cell Proliferation ELISA Kit (abcam, USA) was used for the alternative method of cell proliferation measurement. Cells were seeded at a density of 40,000 cells per well on a 96-well plate and BrdU labeling was performed as per manufacturer's instructions. The CellTiter 96® Non-Radioactive Cell Proliferation Assay (Promega) was used for the measurement of cell number. Cell Death Detection ELISA plus (Roche) was used to measure apoptosis.

ESM method

Cell cycle analysis Cells were seeded at a density of 150,000 cells per well on a 6-well plate and starved on day two in DMEM medium containing 0.1% BSA for 48 hours. Cells were then stimulated with PDGF-BB for 24 hours and trypsinized. The cells were washed once in PBS and fixed in cold ethanol. Propidium iodide was added prior to flow cytometry analysis.

Migration assays Migration was measured using CytoSelect™ 24-Well Cell Migration Assay kit (Cell Biolabs, Inc). 4000 cells were seeded into the Transwell insert and starved on day two in DMEM medium containing 0.1% BSA for 24 hours. PDGF-BB was added for 8 hours and migrated cells were stained and quantified at 560nm as per manufacturer's instructions. Control (control without growth factor stimulation) was set as 1-fold and VSMC migration is presented as relative fold change.

Quantitative RT-PCR Analysis

Total RNA was isolated and purified by RNAeasy plus kit (Qiagen Inc., Valencia, CA) and cDNA was synthesized using high capacity cDNA synthesis kit (Applied Biosystems, Foster City, CA). The qRT-PCR primers used were mouse *Shp-1* Forward 5' -AGA GAA CAA GAG CAA GAA CCG-3'; Reverse 5' -GTT CTC ATC TGG ACC TAG CAG-3'. mouse *Shp-2* Forward 5'–AATGACTTCTGGCGGATGG-3'; reverse 5' - TGACCCCGTATTCTTTGAGC-3'; mouse *Ptp1b* Forward 5' - ACAGTACGACAGTTGGAGTTG-3'; Reverse 5'- TGGTGTAGTGGAAATGCAGG-3'; mouse *Cyclin e1* Forward 5' - GCGAGGATGAGAGCAGTTC-3'; Reverse 5'-AAGTCCTGTGCCAAGTAGAAC-3'. mouse *Mcp-1* Forward 5'–ACCAGCAGCAGGTGTCCCAAAG-3'; reverse 5' -TGGGGTCAGCACAGACCTCTCTC-3'.Mouse *36B4* Forward 5'-GCTCCAAGCAGATGCAGCA-3'; Reverse 5' -CCGGATGTGAGGCAGCAG-3'. qRTPCR was performed using SYBR Green Master mix kit and detection was performed using the ABI PRISM 7900 Sequence Detection System (Applied Biosystems, Foster City, CA). The calculation of relative change in mRNA was performed using the delta-delta method and normalized to house-keeping gene 36B4.

Western blotting

Protein samples were electrophoresed in Bio-Rad® TGX precast gels and transferred to Nitrocellulose membrane, which was blotted with 5% non-fat dry milk in PBS/0.1% Tween-20, and incubated with primary antibody in 4°C overnight. Detection was carried out using Pierce ECL Western Blotting Substrate. Quantitative densitometry was performed using Image J. SHP-1 (sc-287), actin HRP (sc-1616), PDGFRβ (sc-1627), insulin receptor β (sc-711) antibodies were purchased from Santa Cruz. SHP-1 (3759S), SHP-2 (3752), phospho Akt (ser473) (4060L), phospho Erk1/2 (9101L), Akt (9272S), Erk (4695S), PDGFRβ (4564), insulin receptor β (3020s), phospho SAPK/JNK (9251), phospho PDGFRβ Tyr771 (3173) and Tyr751 (3161) were purchased from Cell Signaling. phosphotyrosine (clone 4G10, #05-321) antibody was from Millipore. For Western blotting phospho-Akt and Akt (same molecular weight protein), they were detected on two separate gels instead of stripping membrane to reprobe the other to avoid the inefficiency of stripping. Same techniques were applied to Western blotting phospho-Erk and Erk.

ESM method

Immunofluorescent staining

Mouse femoral arteries were fixed in 4% paraformaldehyde and paraffin embedded. 5 μm sections were made and stained with Ki-67 (abcam, #ab16667), αSMA (Sigma, #A2547), MAC2 (BD Biosciences, clone M3/38 #MABT51), SHP-2 (abcam, #ab ab131541) and PTP1B (abcam, #ab189179) were performed. For the quantification of SHP-1 expression levels in the media of arteries, Photoshop is used for quantifying the intensity of SHP-1 staining. For the Ki-67 and macrophage (MAC2) staining, positive cells with the overlayed of Ki-67 or MAC2 (red) with smooth muscle cell marker (green) and DAPI (nucleus) were counted.

Icariside II oral gavaging in HFD fed mice

All studies with mice were approved by Institutional Review Board at Joslin Diabetes Centre. Icariside I (Syd Lab) was dissolved in 15% PEG 400 (Sigma) and given to the HFD fed mice via daily oral gavaging. 15% PEG 400 was given to vehicle treated mice. Treatment started one day before femoral artery injury.

Statistics

Comparisons of the two groups were made using unpaired t-test. Comparison among more than two groups was performed by one-way ANOVA followed by the post hoc analysis with unpaired t test to evaluate statistical significance between the two groups. The data are mean±SEM, unless otherwise stated. Statistical significance was defined as $p<0.05$.

ESM Table 1: Physiological characteristics of C57BL/6J mice fed on RD and HFD

	RD	HFD (4 wks)	HFD (8 wks)
Body weight (g)	26.1±1.9	34.7±1.3*	38.2±3.9***
Total cholesterol (mmol/L)	2.25±0.10	4.00±0.22***	3.34±0.12***
Insulin (pmol/L)	80.89±6.88	263.31±48.19*	220.29±27.54***
Triglyceride (mmol/L)	0.49±0.05	0.55±0.01	1.04±0.08***

Data are Mean ± SEM. *** $p<0.0001$, * $p<.05$ HFD vs. RD mice; N=4-6 in each group.

ESM Table 2: Patients Clinical Characteristics

	Age (years)	Male	HbA1c (mmol/mol)	Total chol (mM)	LDL (mM)	Trigs (mM)	HDL (mM)	BMI	SBP (mmHg)	DBP (mmHg)
Nondiabetic	71±3	66.70%	35.8±5.3	3.33±0.37	1.42±0.21	1.11±0.24	1.44±0.52	28.17±1.32	139±6	76±5
T2DM	67±2	83.33%	56.6±14.7*	4.73±1.41#	2.63±0.83*	2.48±0.45*	1.17±0.30	30.05±1.67	143±3	76±6

Data are mean ± SEM. N=6 patients in each group. * $p<0.05$ vs. nondiabetic patients; † $p=0.06$ vs. nondiabetic patients. T2DM, type 2 diabetes patients.

ESM Fig. 1

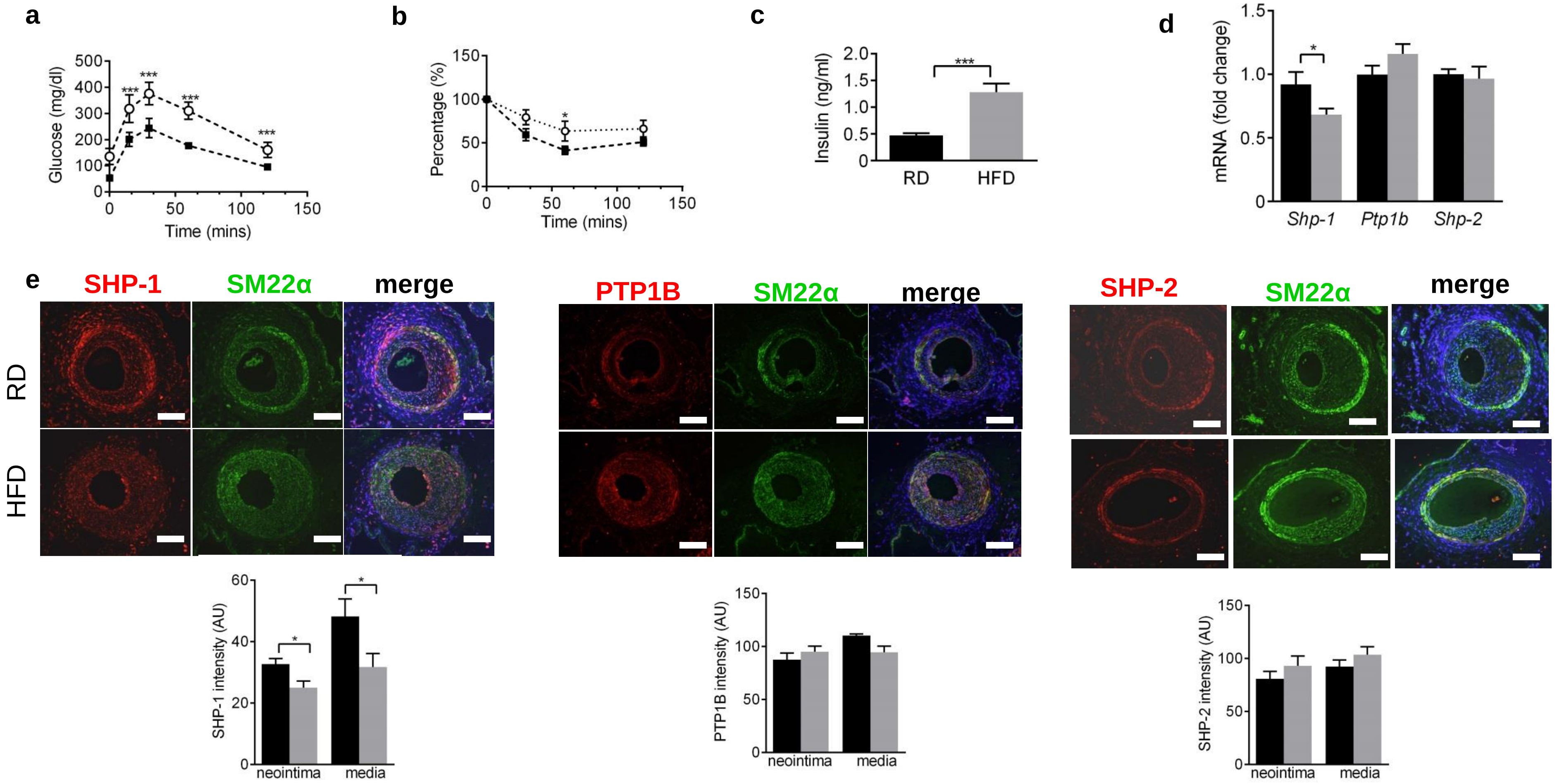

ESM Fig. 1. Metabolic characteristics and different expression levels of PTPs in mice on HFD feeding for 8 wks

(a) Glucose tolerance test (IP-GTT) was performed by i.p. injection of 1mg/g body weight of D-glucose. N=6 mice per group.

(b) Insulin intolerance test (IP-ITT) was performed by i.p. injection of 0.75U per kg body weight of insulin. RD, N=5 mice; HFD, N=7 mice. Black rectangle=RD, white circle=HFD.

(c) Plasma insulin level in HFD feeding for 8 wks. N=6 mice per group.

(d) mRNA levels in femoral arteries from RD and HFD mice on 8 wks HF feeding; N=4 mice per group.

(e) PTPs immunostainining in femoral arteries from RD and HFD mice in response to injury. All merge images were co-stained with Dapi. N=4 mice per group. Scale bar=20 μm.

Black bars=RD, grey bars=HFD. The data are mean ± SEM. ***$p<0.001$, p*<0.05.

ESM Fig. 2

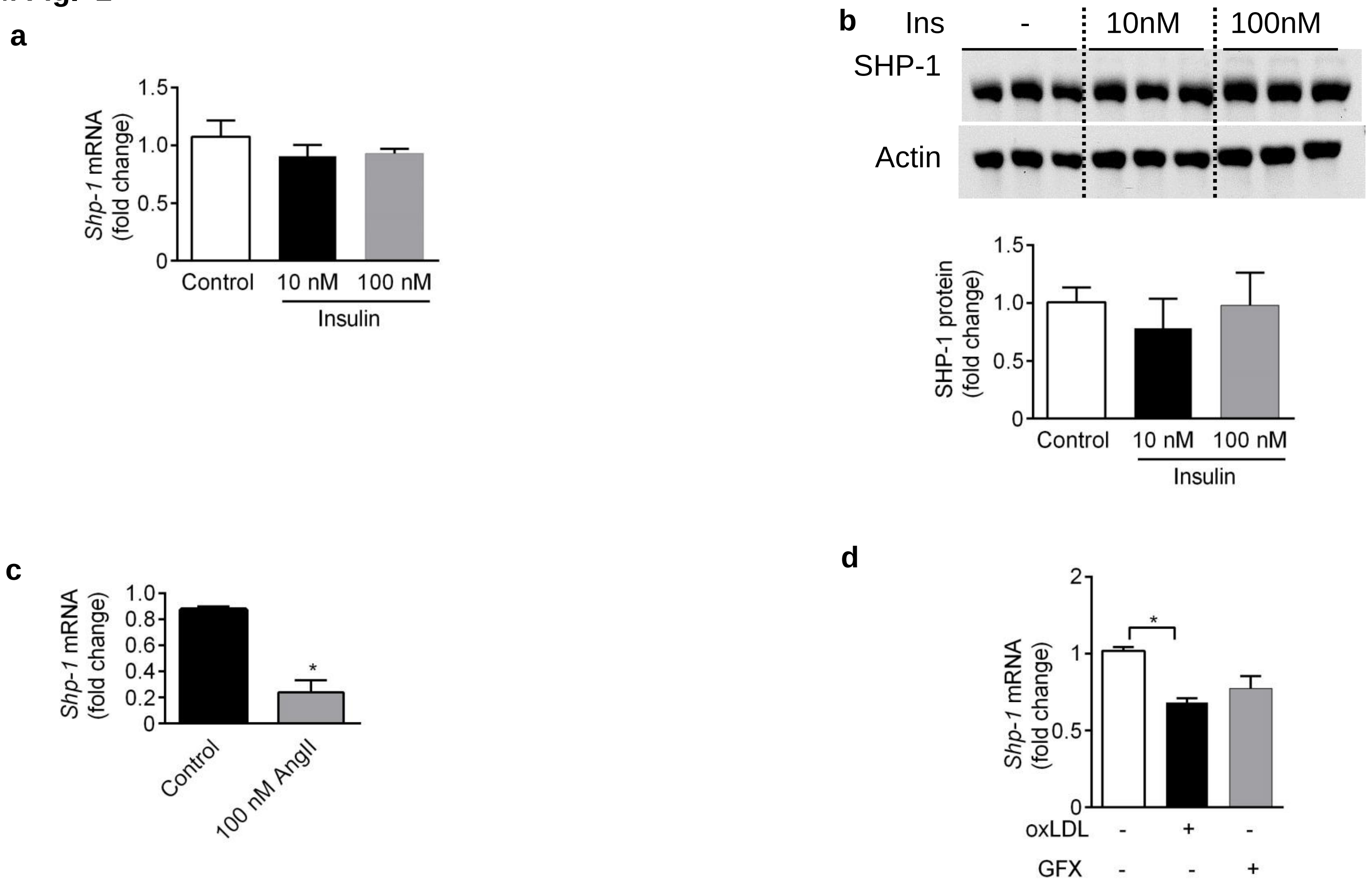

ESM Fig. 2. Effects of insulin, angiotensin II and PKC inhibitor GFX on *Shp-1* expression

VSMC were starved for 24 h in serum free medium containing 0.1% fatty acid free BSA and then **(a)** treated with control (5.6 mmol/l D-glucose) and 10, 100 nmol/l insulin for 8h and *Shp-1* mRNA was measured.

(b) VSMC were treated with control (5.6 mmol/l D-glucose) and 10, 100 nmol/l insulin for 24h.

(c) control (5.6 mmol/l D-glucose) and 100 nmol/l angiotensin II for 8h.

(d) VSMC were treated with control and 100µg/ml oxLDL in the presence or absence of 5 µmol/l PKC inhibitor GFX for 8 hours. The data are mean ± SEM. N=3 independent experiments per group. *p<0.05.

ESM Fig. 3

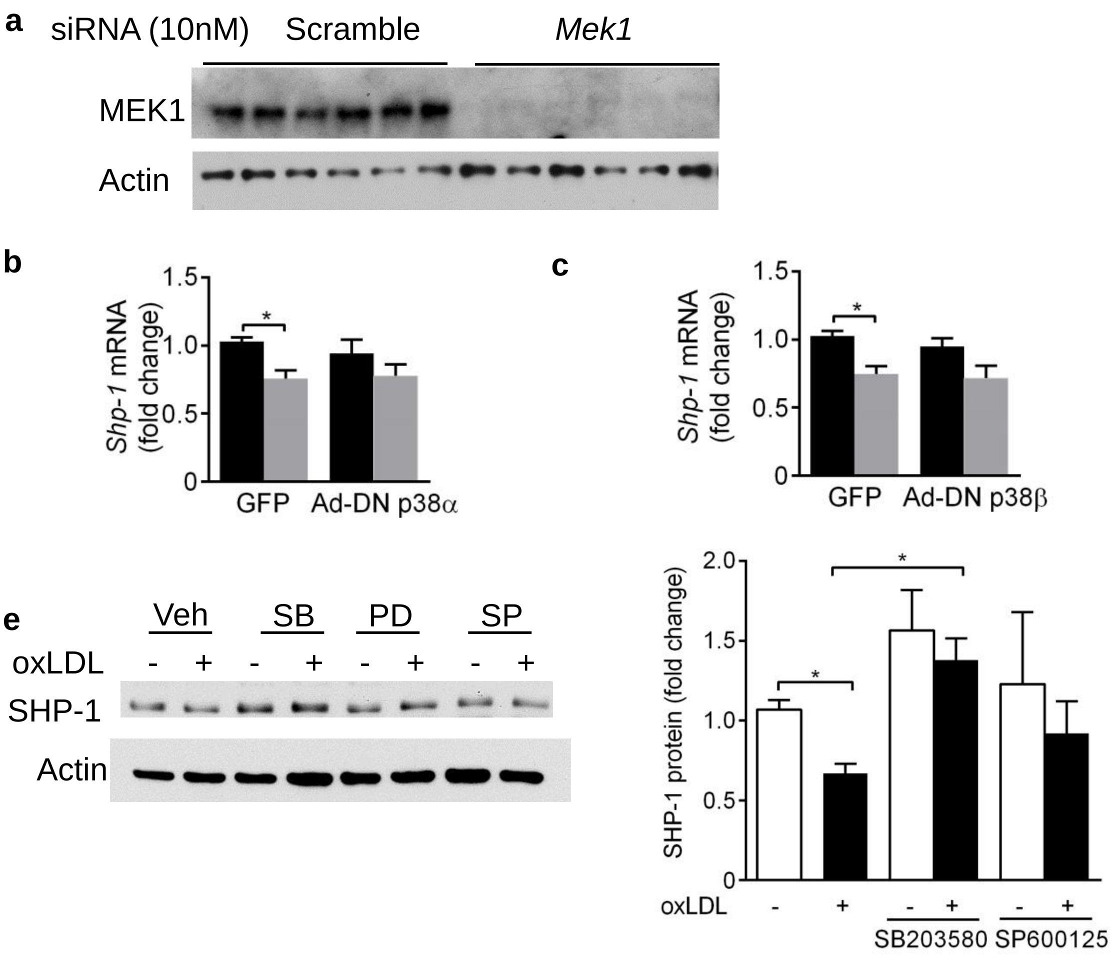

ESM Fig. 3. The roles of MEK1, p38MAPK and JNK pathways in the regulation of SHP-1 by oxLDL

(a) VSMC were transfected with 10nM scramble or *Mek1* siRNA using Lonza SMC kit. MEK1 western blotting was performed.

(b-d) VSMC were infected with dominant negative (DN) adenoviruses of p38MAPKα and β or were transfected with 10nM scramble or *Jnk* siRNA using Lonza SMC kit.. Post-48h infection or transfection, VSMC were treated with 100 µg/ml oxLDL for 8h before RNA were collected for RT-qPCR. Black bars=Control, grey bars=oxLDL.

(e) VSMC were treated with control (5.6 mmol/l D-glucose) and 100µg/ml oxLDL in the presence or absence of 10 µmol/l SB (SB203580) and SP (SP600125) for 24 hours. The data are mean ± SEM. N=5 independent experiments per group. ***p*< 0.01; **p*<0.05.

ESM Fig. 4

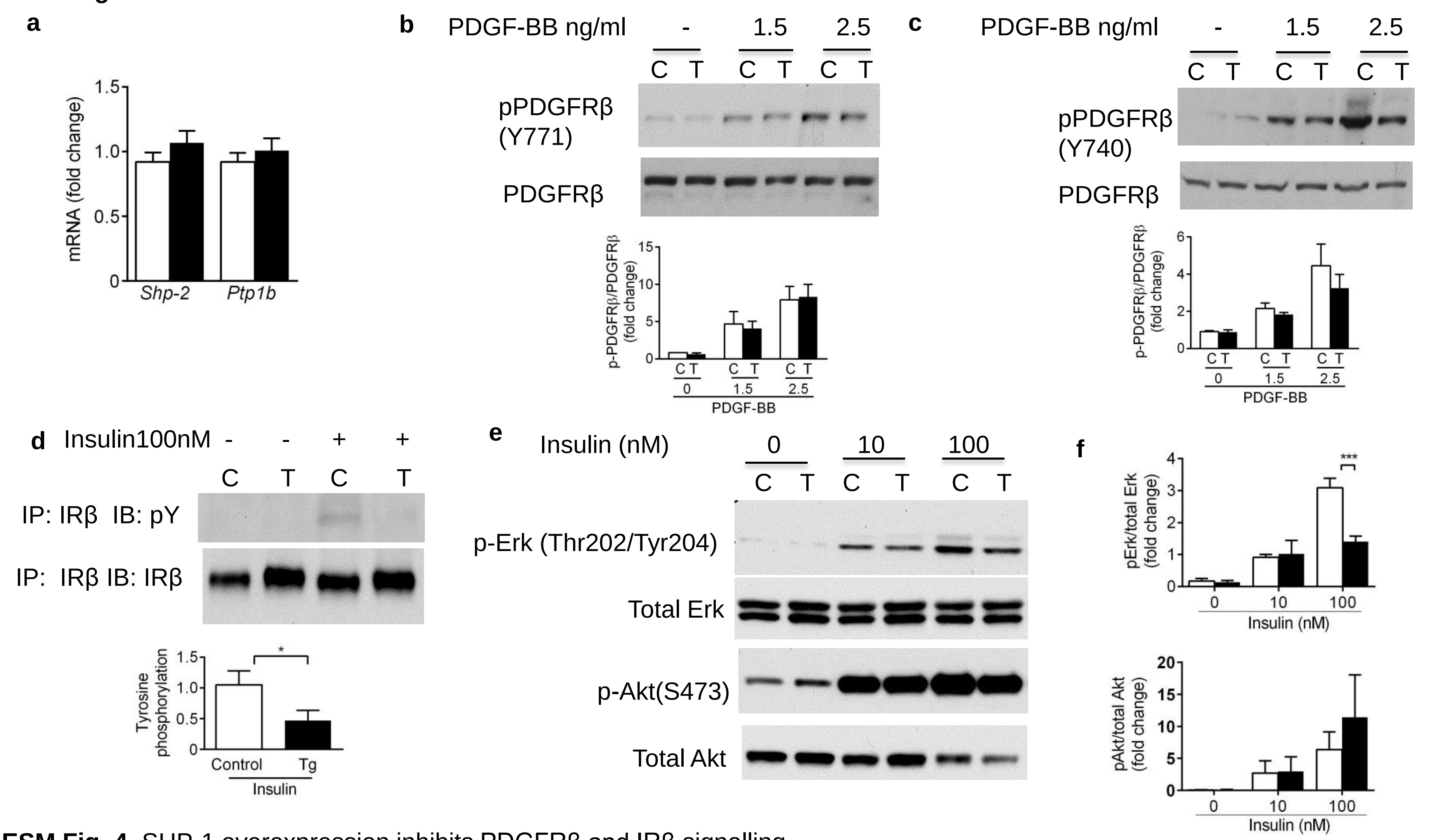

ESM Fig. 4. SHP-1 overexpression inhibits PDGFRβ and IRβ signalling
(a) mRNA expression in VSMC from Control and SHP-1Tg mice. The data are mean ± SEM. N=5 independent experiments per group. **(b, c)** VSMC was stimulated with PDGF-BB for 5 mins. Y771 and Y740 were quantified by Western blotting. N=4 independent experiments per group. **(d)** VSMC was stimulated with 100nmol/l insulin for 5 mins and cell lysate were harvested for co-immunoprecipitation with insulin receptor β (IRβ) antibody. pTyr and IRβ Western blotting was subsequently performed. The data are mean ± SEM. N=3 independent experiments per group. **(e-f)** VSMC was stimulated with 10 and 100nmol/l insulin for 5 mins. p-Erk, p-Akt, total Erk and Akt Western blotting were performed. The data are mean ± SEM. N=3 independent experiments per group. White bars=Control (C), black bars=Tg (T). ***$p<0.001$; *$p<0.05$.

ESM Fig. 5

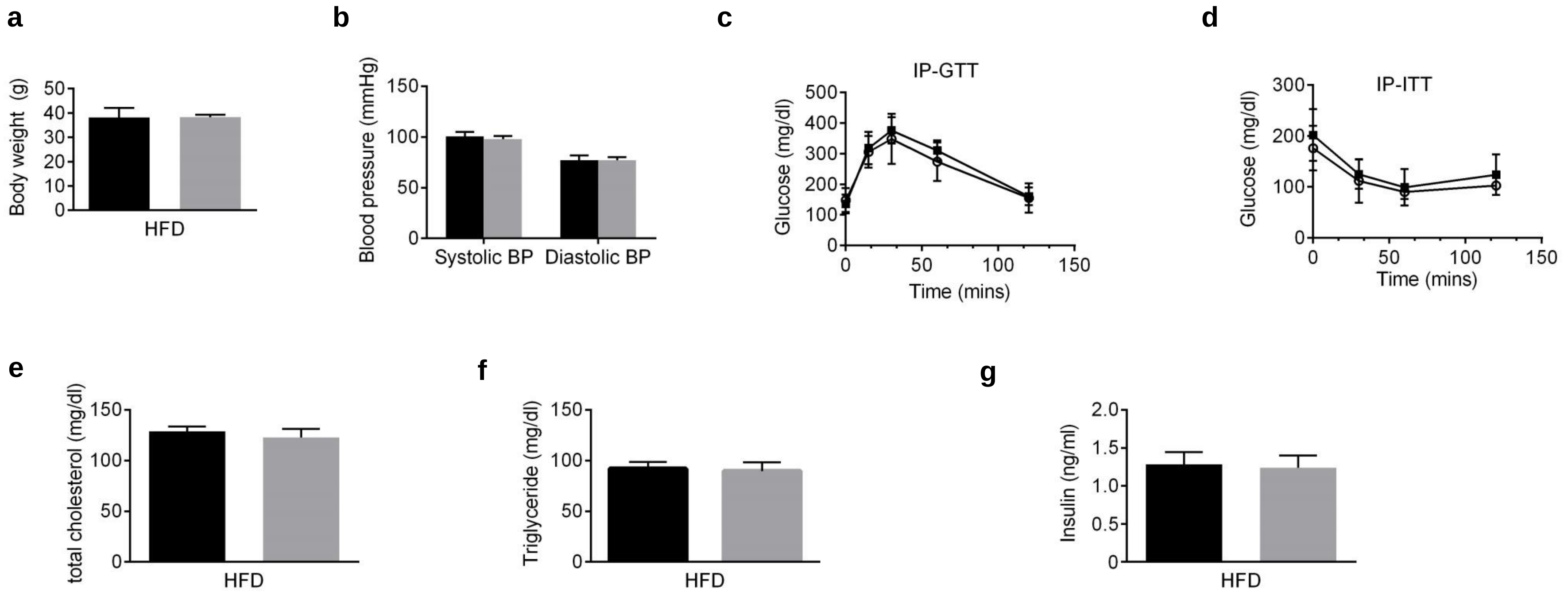

ESM Fig. 5: Metabolic characteristics of VSMC-specific SHP-1 overexpression mice on HFD
Control and Tg mice were fed on HFD for 8wks. The data are mean ± SEM. N=4-10 mice per group for the metabolic measurements. **(a)** Body weight (BW). **(b)** Systolic and diastolic blood pressure were measured using tail-cuff method; **(c)** IP-GTT was performed by i.p. injection of 1mg/g body weight of D-glucose. **(d)** IP-ITT was performed by i.p. injection of 0.75U per kg body weight of insulin. Black rectangle=Control HFD, white circle=Tg HFD. Total cholesterol **(e),** triglyceride **(f)** and insulin **(g)** were measured. Black bars=Control, grey bars=Tg.

ESM Fig. 6

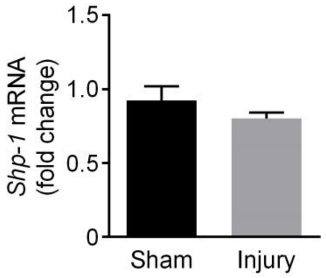

ESM Fig. 6: SHP-1 expression in response to injury
Shp-1 mRNA was measured in femoral arteries from sham (n= 5) and post-9 days wire injury (n=7) mice. The data are mean ± SEM.

ESM Fig. 7

a

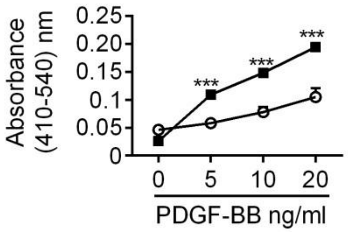

b

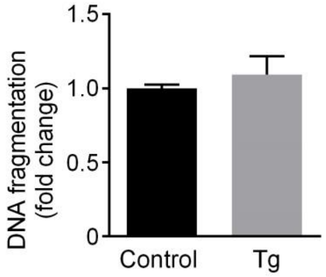

ESM Fig. 7: Overexpression of SHP-1 on cell proliferation and apoptosis in VSMC

(a) VSMC were stimulated with different doses of PDGF-BB (0, 5, 10, and 20ng/ml) for 24h. BrdU proliferation kit was used and read at 410-540nm. The data are mean ± SEM. N=4 independent experiments per group. ***p<0.001 vs. control (no PDGF-BB). Black rectangle=Control, white circle=Tg.

(b) VSMC were incubated with 0.1%BSA for 24h. DNA fragmentation in Control (n=4) and SHP-1Tg (n=6) VSMC. The data are mean ± SD.
